# Clinical Prospect of Mesenchymal Stromal/Stem Cell-Derived Extracellular Vesicles in Kidney Disease: Challenges and the Way Forward

**DOI:** 10.3390/pharmaceutics15071911

**Published:** 2023-07-09

**Authors:** Maja Kosanović, Bojana Milutinović, Tanja J. Kutzner, Yanis Mouloud, Milica Bozic

**Affiliations:** 1 Institute for the Application of Nuclear Energy (INEP), University of Belgrade, 11 000 Belgrade, Serbia; maja@inep.ac.rs; 2 Department of Neurosurgery, MD Anderson Cancer Center, University of Texas, Houston, TX 770302, USA; bmilutinovic@mdanderson.org; 3 Institute for Transfusion Medicine, University Hospital Essen, University of Duisburg-Essen, 45355 Essen, North Rhine-Westhpalia, Germany; tanjajasmin.kutzner@uk-essen.de (T.J.K.); yanis.mouloud@uk-essen.de (Y.M.); 4 Vascular and Renal Translational Research Group, Biomedical Research Institute of Lleida Dr. Pifarré Foundation (IRBLLEIDA), 25196 Lleida, Spain

**Keywords:** mesenchymal stromal/stem cells, extracellular vesicles, kidney disease, acute kidney injury, chronic kidney disease, transplantation, renoprotection, cell-free therapeutics, mechanism of action, clinical application

## Abstract

Kidney disease is a growing public health problem worldwide, including both acute and chronic forms. Existing therapies for kidney disease target various pathogenic mechanisms; however, these therapies only slow down the progression of the disease rather than offering a cure. One of the potential and emerging approaches for the treatment of kidney disease is mesenchymal stromal/stem cell (MSC) therapy, shown to have beneficial effects in preclinical studies. In addition, extracellular vesicles (EVs) released by MSCs became a potent cell-free therapy option in various preclinical models of kidney disease due to their regenerative, anti-inflammatory, and immunomodulatory properties. However, there are scarce clinical data available regarding the use of MSC-EVs in kidney pathologies. This review article provides an outline of the renoprotective effects of MSC-EVs in different preclinical models of kidney disease. It offers a comprehensive analysis of possible mechanisms of action of MSC-EVs with an emphasis on kidney disease. Finally, on the journey toward the implementation of MSC-EVs into clinical practice, we highlight the need to establish standardized methods for the characterization of an EV-based product and investigate the adequate dosing, safety, and efficacy of MSC-EVs application, as well as the development of suitable potency assays.

## 1. Introduction

Kidney disease is a serious health concern worldwide with an increasing prevalence [1]. Growing evidence suggests that even minor types of kidney disease are linked to an elevated risk of developing kidney failure, as well as a higher likelihood of cardiovascular disease (CVD) and death [1]. While there have been some improvements in understanding the pathophysiology of kidney disease, current treatments are limited in their ability to halt or reverse disease progression. Therefore, the development of new strategies for treating kidney disease is a critical area of research. 

Stem cell therapies have shown potential in the treatment of different diseases and have been tested in preclinical models and clinical trials. In particular, the use of extracellular vesicles (EVs) derived from stem cells has emerged as a promising new treatment option for promoting recovery from various types of injuries. In this context, EVs derived from mesenchymal stromal/stem cells (MSCs), known as MSC-EVs, have become a potent cell-free therapy for several disease states, including kidney disease [2]. MSC-EVs can modulate immune responses, promote tissue repair, and inhibit cell death [3]. Due to their therapeutic potential and the ease of harvesting, MSC-EVs are emerging as a promising new drug for the treatment of kidney disease and several other disease states.

This review article provides a summary of the renoprotective effects of MSC-EVs in various preclinical models of kidney disease and offers details on the possible mechanisms of action (MoA) of MSC-EVs. As we progress towards the clinical use of MSC-EVs, the article underscores the importance of creating standardized methods for the characterization of EV-based products, studying the appropriate dosing, safety, and efficacy of MSC-EVs administration, and developing suitable potency assays.

## 2. Kidney Disease: Definition and Classification 

Kidney disease is described as a heterogeneous group of disorders that affect kidney structure and function [1]. An increasing amount of evidence implies that even moderate forms of kidney disease are linked to a higher risk of kidney failure, as well as the risk of cardiovascular morbidity and death [1,4]. Based on the progression of the injury, kidney diseases are stratified into acute kidney injury (AKI), chronic kidney disease (CKD), and end-stage kidney disease (ESKD). 

AKI is defined as an abrupt decrease in kidney function [5] and represents a serious clinical problem. It has been reported that around 22% of adults and 34% of children across the world experience AKI during hospitalization [6]. Despite major advancements in preventive measures and supportive care, AKI continues to be linked to higher rates of morbidity and mortality [3], where mortality rates for intensive care unit patients can exceed 50% [5]. Numerous stressors including ischemia, nephrotoxic medications, environmental pollutants, nephritis, and urinary tract obstruction [5] can cause an injury of cells within the nephron, specifically kidney proximal tubular cells, leading to cell damage, inflammation, oxidative stress, apoptosis, and vascular dysfunction [7]. Depending on the degree of kidney damage, AKI can lead to regeneration and recovery of the injured tissue, or the CKD, known as AKI-to-CKD transition [8,9]. Many patients who survive their acute illness fail to recover normal kidney function and develop CKD and ESKD [10]. The relationship between AKI and CKD is very complex: AKI can lead to CKD, and CKD increases the risk of AKI [4]. 

CKD is known as one of the major noncommunicable diseases worldwide, and up to 10% of the world’s adult population is assumed to be affected by this condition [4], with increasing prevalence in the aging population [11,12]. According to the National Kidney Foundation’s Kidney Disease Outcomes Quality Initiative (NKF-KDOQI), CKD is classified into stages 1–5 [13]. In the early stages of CKD, 1 and 2, kidney function is still in the compensatory period with mild changes in proteinuria and glomerular filtration rate (GFR) [14]. As the disease progresses, the kidney function deteriorates and the patient enters stages 3 and 4 of CKD, with the loss of about 50% of normal kidney function [14]. Stage 5 is the last stage of CKD and is characterized by a GFR of less than 15 mL/min/1.73 m^2^ or the requirement for maintenance dialysis [13,14]. The main pathological changes associated with CKD include kidney interstitial fibrosis and glomerulosclerosis; while kidney fibrosis is considered to be the final manifestation of all CKDs regardless of their etiology [15,16,17]. 

ESKD sets up when CKD reaches an advanced stage and kidneys lose their filtering abilities (GFR < 15 mL/min/1.73 m^2^). Patients with ESKD have a high mortality risk, with CVD mortality accounting for 53% of deaths [18]. At this stage, renal replacement therapies (mainly hemodialysis and peritoneal dialysis) or a kidney transplant are needed to prolong patients’ lives. 

Current therapeutic strategies for AKI and CKD focus on supportive care to manage complications and slow disease progression; however, they are insufficient to reverse disease progression. The development of new approaches that enable the restoration of damaged kidney tissue or prevent the advancement to CKD is crucial in improving short-term and long-term outcomes. 

## 3. Extracellular Vesicles—A New Paradigm Shift in the MSC Therapy 

Numerous studies have confirmed the beneficial effects of MSCs for the treatment of a variety of pathologies, including kidney diseases [19,20,21,22,23,24]. Nevertheless, in the MSC field, it became obvious that MSCs exert therapeutic functions via their secretome [25,26,27,28], and that the biologically active component of MSC secretome, i.e., extracellular vesicles (EVs), can be used in place of MSCs as potential therapeutics. 

EVs comprise a heterogeneous group of membrane-enveloped vesicles, that are secreted by cells into the extracellular space via different routes. According to classical nomenclature, EVs are divided into three main types: (a) exosomes, formed by the invagination of the limiting membrane of the early endosome, i.e., multivesicular body (MVB) and released into the extracellular space upon fusion of MVB with the plasma membrane; (b) microvesicles (MVs), formed by outward budding and detachment from the plasma membrane; and (c) apoptotic bodies, released during programmed cell death. However, in recent years, several other types of EVs released from plasma membranes have been discovered such as migrasomes, ciliary ectosomes, secreted midbody remnants, exophers, etc., which E.I. Buzas, in her recently published paper, classified into two major categories: exosomes (originating from endosomal compartment) and ectosomes (originating from plasma membrane) [29]. 

EVs contain luminal, transmembrane, and membrane-associated molecules, including proteins, nucleic acids, lipids, and metabolites. The diameter of EVs falls in the nanometer range (from 30 nm to several hundreds of nanometers), with exosomes being considered as generally smaller than some classes of ectosomes (i.e., microvesicles or apoptotic bodies). The size of EVs considerably overlaps between types, rendering size an unreliable criterion for distinguishing them [29,30]. Furthermore, there are no specific markers of EV type, although some tetraspanins (e.g., CD9, CD63, CD81) are used as EV markers, with CD81 being more enriched in small EVs (including exosomes) and CD9 in larger EVs (including MVs) [31]. The lack of differential markers, as well as overlap in other physical or biochemical properties, cause a shortfall of techniques able to isolate EVs of just one type. Consequently, the International Society of Extracellular Vesicles (ISEV) recommends using the general term “extracellular vesicles” for any vesicles isolated from body fluids or cell culture media, with guidelines for the application of biomarkers for in-depth characterization [30,32,33]. However, it is important to notice that nomenclature varies throughout the literature, especially for the one published before the release of ISEV guidelines [30,34], as well as in some recent publications. The term “exosomes” is still used by some researchers and also in the industry to designate all EVs. Since this inaccuracy in the utilization of the nomenclature can lead to misinterpretation of results, it is crucial for every reader to critically evaluate methods applied for separation of EVs and biochemical characterization used in the literature sources, in order to compare results. In this review, we will use the term “extracellular vesicles” and EVs regardless of the literature source, in order to better align original data with the current understanding of EVs biology.

EVs have the ability to transfer complex information or signals from one cell to another in a targeted manner, affecting the biology and function of the recipient cell. Theoretically, EVs can transmit the information through (a) internalization of EVs by target cells through endocytosis; (b) fusion of EVs with the target cell membrane; or (c) interaction of EV´s surface molecules with the recipient cell. The mode of information transfer, the type of the EV interaction with the recipient cell, and the type of active molecules depend on the EV type and likely specific subpopulation, i.e., on their content and characteristics of the recipient cell. However, we still lack in-depth knowledge about subpopulation heterogeneity to precisely attribute specific mechanisms to each subpopulation. Thus, although there are a plethora of information about the importance of both proteins and RNA [35,36,37,38,39] in the transfer of information by EVs, there are some studies that reveal subpopulations of MSC-EVs without RNA [40] or re-evaluate the influence of RNA [41,42], emphasizing the need for further exploration of the heterogeneity of EVs and elucidation of their mechanisms using advanced methodological approaches. 

Nevertheless, the transfer of information by EVs is a universal mechanism by which cells communicate and they are involved in all physiological and pathological processes investigated so far [43]. Hence, the characteristics of EVs reflect the physiological state of the originating cells, and EVs can be used as a potential novel class of biomarkers [44,45,46]. Also, EVs are both targets and tools of novel therapeutic approaches, such as anti-tumor therapy, vaccination, modulation of the immune system, and drug delivery [2,25]. A proof of concept showing that MSC-EVs can be used therapeutically in humans was first demonstrated in 2014 when they were used as a treatment for a refractory acute Graft-versus-Host disease (GvHD) patient, whose symptoms improved significantly during a 2-week-long treatment period and remained stable for more than four months [47].

MSC-EVs are also considered a good therapeutic option due to several important advantages in comparison to MSC cellular therapy. Therapeutic MSCs need to be expanded in the culture, often under different conditions, conferring phenotypic differences to the final product [48]. Possible cellular senescence may lead to a loss of therapeutic properties [49]. Certain concerns are related to the probability of cellular rejection, formation of ectopic tissue, or embolism in small lung blood vessels [50]. It is accepted that MSC-EVs can be produced in large quantities and stored for prolonged periods without significant changes in biological properties. Additionally, MSC-EVs are not self-replicating, and their small size eliminates the risk of embolism. In addition to the clinical application of EVs in GvHD patient, results have been reported for the treatment of CKD patients, a patient obtaining a cochlea implant, and severe COVID-19 patients [51,52,53]. None of these studies reported side effects, while positive impacts of the MSC-EVs application were described in all studies. MSC-EVs have been shown to mediate immunomodulatory, anti-inflammatory, and pro-regenerative activities [48], qualifying them as a potential therapeutic option for a variety of diseases.

## 4. Demonstrable Renoprotective Effects of MSC-EVs in Preclinical Studies of Kidney Disease

The beneficial effects of MSC-EVs in ameliorating kidney damage have been shown in various preclinical models of AKI and CKD, thus demonstrating their potential value in the treatment of kidney failure. In these studies, EVs derived from MSCs isolated from different adult and neonatal sources such as bone marrow, adipose tissue, liver, kidney, umbilical cord, and placenta, have been used (Table 1).

Bone marrow (BM)-derived MSC-EVs (BM-MSC-EVs) exert beneficial effects in the renal ischemia-reperfusion (I/R) injury (IRI) model of AKI [54,55,56] and CKD [57]. The treatment of mice or rats with BM-MSC-EVs reduced tubular lesions and fibrosis; decreased lymphocyte infiltration; and enhanced tubular cell proliferation. Meanwhile, at the functional level, they lowered blood urea nitrogen (BUN), creatinine, and proteinuria. In AKI caused by glycerol, cisplatin, or gentamicin, similar effects were noted [27,58,59,60]. Namely, BM-MSC-EVs were able to restore the brush border and increase mitochondria, inhibit necrosis, apoptosis, and inflammation, enhance proliferation and survival, as well as to reduce the BUN and creatinine levels. Beneficial effects of BM-MSC-EVs have also been demonstrated in models of CKD such as unilateral ureteral obstruction (UUO) [61,62,63,64], nephropathy induced by aristolochic acid (AAN) [65], or diabetic nephropathy (DN) induced by streptozotocin [66]. In all these models, EVs ameliorated kidney damage. In the UOO model, BM-MSC-EVs reduced tubular dilation and interstitial inflammatory cell infiltration; preserved intact cortical tubules; reduced the disruption of the tubular basement membrane and ECM deposition; preserved E-cadherin levels; and decreased serum creatinine, BUN, and proteinuria. In the AAN model, BM-MSC-EVs showed regenerative and anti-fibrotic effects, as well as the anti-inflammatory effect seen as a decrease of the infiltration of CD45 positive immune cells, fibroblasts, and pericytes, similar to in the STZ-DN model [65,66].

**Table 1:** MSC-EVs sources and administration routes in various preclinical models of kidney disease.

EV Source	In Vivo Model	Type of Kidney Disease	Administration	Reference
BM-MSCs	Glycerol	AKI	Intravenous	[27]
	I/R	AKI	Intravenous	[54]
	I/R	AKI	Intravenous	[55]
	Cisplatin	AKI	Intravenous	[58]
	Gentamicin	AKI	Intravenous	[59]
	Glycerol	AKI	Intravenous	[60]
	I/R	AKI	Intravenous	[56]
	UUO	CKD	Intravenous	[61]
	I/R	CKD	Intrarenal	[57]
	AAN	CKD	Intravenous	[65]
	UUO	CKD	Intravenous	[62]
	UUO	CKD	Intravenous	[63]
	STZ-DN	CKD	Intravenous	[66]
	UUO	CKD	Intravenous	[64]
AD-MSCs	I/R	AKI	Intravenous	[67]
	Sepsis	AKI	Intravenous	[68]
	MS+AS	CKD	Intrarenal	[69,70]
	I/R	CKD	Intravenous	[71]
	UUO	CKD	Intravenous	[72]
	Glycerol	AKI	Intravenous	[73]
L-MSCs	STZ-DN	CKD	Intravenous	[66]
	AAN	CKD	Intravenous	[74]
K-MSCs	I/R	AKI	Intravenous	[75]
	I/R	AKI	Intravenous	[76]
	UUO	CKD	Intravenous	[77]
UC-MSCs	Cisplatin	AKI	Kidney capsule	[78]
	I/R	AKI	Intravenous	[79]
	I/R	AKI	Intravenous	[80]
	I/R	AKI	Intravenous	[81]
	I/R	AKI	Intravenous	[82]
	I/R	AKI	Intravenous	[83]
	I/R	AKI	Intravenous	[84]
	Cisplatin	AKI	Kidney capsule	[85]
	I/R	AKI	Intravenous	[86]
	Sepsis	AKI	Intravenous	[87]
	I/R	AKI	Intravenous	[88]
	UUO	CKD	Intravenous	[89]
	I/R	CKD	Intravenous	[86]
	I/R	CKD	Intravenous	[79]
	I/R	CKD	Intravenous	[84]
P-MSCs	I/R	AKI	Intrarenal	[90]
	I/R	AKI	Intrarenal	[91]

MSCs, mesenchymal stromal/stem cells; BM-MSCs, bone marrow MSCs; UC-MSCs, umbilical cord MSCs; AD-MSCs, adipose tissue MSCs; P-MSCs, placental MSCs; K-MSCs, kidney-derived MSCs; L-MSCs, liver MSCs; UUO, unilateral ureteral obstruction; I/R, ischemia-reperfusion; AAN, aristolochic acid-induced nephropathy; STZ-DN, streptozotocin-induced diabetic nephropathy; MS+AS, metabolic syndrome, and renal artery stenosis.

Adipose tissue-derived MSCs (AD-MSCs) are another type of MSCs recognized as a valuable source of EVs potentially therapeutic for kidney diseases. Thus, in the sepsis-induced AKI model, AD-MSC-EVs managed to abrogate inflammation, apoptosis, and microcirculation disorders, lowering overall mortality [68]. In CKD, AD-MSC-EVs were proven to have beneficial effects in models of metabolic renovascular disease and metabolic syndrome with renal artery stenosis [69,70], IRI [71], and UUO [72]. In these models, AD-MSC-EVs alleviated CKD by attenuating kidney inflammation, apoptosis, oxidative stress, and fibrosis, improving medullary oxygenation and GFR. They were also able to influence kidney blood flow by lowering renal artery stenosis or improving cortical microvascular peritubular capillary density.

EVs from liver stem-like cells (HLSCs) have also been investigated for their renoprotective potential in models of glycerol-induced AKI, DN in streptozotocin-induced diabetes and AAN [66,73,74], and they elicited similar effects as EVs from other types of MSCs.

MSCs derived from the kidney itself also produce EVs with the potential to induce beneficial changes in kidney diseases. Thus, in the IRI model of AKI [75,76], those EVs induced improvement in the proliferation of peritubular capillary endothelial cells, microvascular rarefaction, reduction of ischemic damage, and overall amelioration of kidney function. In the UUO model of CKD [77], they inhibited endothelial-to-mesenchymal transition of peritubular capillaries (PTC) endothelial cells, improved PTC rarefaction, and inhibited inflammatory cell infiltration and TIF.

Umbilical cord MSC-EVs (UC-MSC-EVs) have been used in various models of kidney disease. In IRI models [79,80,81,82,83,84,86,88], UC-MSC-EVs were found to mitigate renal cell apoptosis; enhance proliferation and tubular epithelial cell growth; increase capillary vessel density; decrease epithelial cell swelling, necrosis, and casts formation; and abrogate kidney fibrosis, inflammation, and oxidative stress, thus improving kidney function. UC-MSC-EVs have also shown renoprotective effects against cisplatin-induced nephrotoxicity and were able to prevent the development of AKI [78,85]. Their application led to an increase of PCNA-positive cells; reduction of oxidative stress, apoptosis, and necrosis of renal proximal tubules; and improvement of kidney function. In the sepsis model [87], UC-MSC-EVs alleviated sepsis-associated AKI improving the survival of affected mice. UC-MSC-EVs were also applied as treatment in CKD models. In IRI models of CKD [79,84,86], UC-MSC-EVs reduced apoptosis, inflammation, and fibrosis; enhanced proliferation and the number of PCNA-positive cells; increased capillary vessel density; and improved kidney function. Similar effects were observed in the UUO model of CKD [89].

Placental MSC-EVs were tested in IRI models of AKI [90,91] where they increased the proliferation of kidney tubular epithelial cells and angiogenesis, alleviated apoptosis and fibrosis, and improved levels of serum BUN and creatinine. 

As seen from the evidence above, different types of MSC-EVs have the potential to modulate various key aspects of AKI and CKD pathogenesis, improving kidney damage in different preclinical models, which makes them good candidates for the development of novel therapy for kidney disease.

## 5. Utilization of MSC-EVs in Clinical Trials

### 5.1. MSC-EVs in Clinical Trials of Kidney Disease

As seen from a variety of preclinical models, MSC-EVs emerged as a promising agent in promoting recovery and regeneration after kidney injury. However, clinical studies testing the efficacy and safety of MSC-EVs in kidney pathologies are limited. To date, there is only one study assessing the therapeutic effect of MSC-EVs in patients with CKD. Nassar et al. (2016) conducted a randomized, placebo-controlled study investigating the safety and therapeutic efficacy of EVs derived from human cord blood MSCs (CB-MSCs) in alleviating the progression of CKD stages 3 and 4 [51]. Forty patients with diagnosed CKD stages 3 and 4 were enrolled in the study. They were randomly divided into a treatment group consisting of 20 CKD patients receiving CB-MSC-EVs and a control group composed of 20 CKD patients receiving saline. EVs were administered intravenously and intra-arterial with a one-week break between two injections. The authors reported a transient improvement of kidney function in stage 3 and 4 CKD patients, followed by the amelioration of inflammatory immune reaction [51]. Besides the beneficial effects, the administration of MSC-EVs were shown to be safe for the patients enrolled in the study. 

### 5.2. What Can We Learn from MSC´s Clinical Trials?

The therapeutic potential of MSCs has long been recognized and numerous clinical trials are underway. In kidney disease, clinical trials are being conducted in AKI and CKD, DN, lupus-related nephropathy, and kidney transplants (ClinicalTrials.gov, accessed on 25 February 2023). Due to abundant experience in the production and application of clinical/therapeutic MSCs, some common conclusions can be made, and parallels can be drawn to better understand the future directions in the production of clinical-grade EVs. Furthermore, several bottlenecks associated with MSC-derived EVs need to be addressed in order to harness the full potential of MSC-derived EVs for therapeutic application, such as standardization of isolation methods for EVs, scalability and production efficiency, heterogeneity of EV populations, cargo loading methods, storage, and stability. 

#### 5.2.1. Characterization of Product for Clinical Trials

In the early 2000s, numerous reports on the therapeutic use of MSCs brought to our attention the fact that different methods of isolation and expansion of MSCs, as well as different approaches to characterize the cells, made it difficult to compare results of individual studies, underlining the need to define the minimum criteria for defining MSCs. In a position paper, the Mesenchymal and Tissue Stem Cell Committee of the International Society for Cellular Therapy proposed that MSCs must (1) be plastic-adherent under standard culture conditions; (2) express CD105, CD73, and CD90, and lack expression of CD45, CD34, CD14, or CD11b, CD79α or CD19, and HLA-DR surface molecules; and (3) differentiate into osteoblasts, adipocytes, and chondroblasts in vitro [92]. However, these criteria are insufficient for predicting the therapeutic potency of MSCs and do not consider factors such as cell source, culturing conditions, intrinsic donor variability, and effects of microenvironment after infusion (reviewed in [93]), contributing to the failure of some later stage clinical trials. Additionally, therapeutic MSCs are tested for viability, sterility, endotoxin, and mycoplasma before being released into the clinical setting [94,95,96,97]. 

In comparison, the characterization of EVs for clinical trials meets different challenges. Even though criteria for immunophenotypic characterization are defined [30], characterization at the single-vesicle level requires the use of advanced-flow cytometry [98]. Ensuring high purity of preparation is very important: both exogenous EVs (from fetal bovine serum (FBS) or human platelet lysate (hPL)) and non-EVs components (including protein, RNA, and lipid) may confound the therapeutic potential of MSC-EVs. An additional characterization of MSC-EVs based on specific lipid content may improve the reliability of data [30]. Implementation of rigorous criteria for phenotypic characterization of EVs using flow cytometry and the development of nanoflow technologies will increase the standard of production of clinical-grade EVs [98]. 

#### 5.2.2. Dosing and Safety

The therapeutic dose of MSCs is commonly established as the number of cells per body mass [94,95,96,97]. In assessment of safety, MSC clinical study design estimates the increase of the treatment dose, while simultaneously monitoring for adverse effects (CTCAE) and dose-limiting toxicity (DLT). The study proceeds with the highest dose in which the adverse effects were not observed. 

In comparison to MSCs, parallel attempts to define MSC-EVs for clinical trials fall short, due to limitations of technology used to quantify and characterize EVs. Quantification of EVs using nanoparticle tracking analysis (NTA), dynamic light scattering (DLS), or tunable resistive pulse sensor (TRPS) is hampered by the lack of discrimination between small EV and non-EV particles, including lipoproteins and protein aggregates. A recent position paper on the development of EV-based medicinal products, recommends the use of these technologies in combination with immune-chemical characterization [99]. 

Safety concerns in the application of MSCs and MSC-EVs are somewhat different. While in the case of MSC-based therapies, phenotypic stability (and related loss of therapeutic potential) present major concern, in the case of EVs, these concerns are related to the mechanism of action (MoA) and the target identity, which are often insufficiently understood [99]. 

Clinical trials of MSCs, performed to treat kidney disease, demonstrated that MSCs can be safely given to humans and are generally well tolerated [94,95,96,97] with no difference in the rate of adverse effects between groups treated with MSCs and placebo [100]. Taking this into account and considering (native) EVs are physiologically produced and taken up in different tissues, there is little expectation of adverse events. However, adverse effects may arise from events related to anticipated MoA, and they should be closely monitored and reported [99].

#### 5.2.3. Efficacy 

In terms of efficacy, available data require careful consideration with respect to pathology, endpoints used to monitor patient outcomes and differences in study design. Several, early-phase clinical trials used MSCs to treat AKI associated with cardiac surgery. Studies that used MSCs concurrent with surgery in patients with increased risk of AKI, reported shorter hospital stays [101,102]. However, when AKI developed during cardiac surgery, subsequent MSCs treatment did not decrease the time necessary for the recovery of kidney function [100]. In CKD, studies reported a significant decrease in unfavorable outcomes compared to the placebo group [103,104]. However, when parameters of kidney function are monitored, results may be conflicting: the improvement may be recorded based on protein excretion rate [105], but the lack of effect was reported when GFR is monitored [105,106]. More consistent efficacy was reported in lupus, with studies reporting stabilization of kidney function in systemic lupus erythematosus (SLE) [107,108,109] and long-term stabilization of proteinuria in lupus nephritis (LN) [110]. However, at least one clinical trial showed no differences between MSCs treatment and standard immunosuppressive therapy [111]. 

These data show that a careful choice of endpoints is a prerequisite for the success of phase III clinical trials which are intended to show treatment efficacy. Combining several endpoints, based on the proposed MoA, may help improve the outcome of clinical trials. This also underlines the importance of an in-depth understanding of MoA, which is often lacking when the therapeutic effects of EVs are considered. 

#### 5.2.4. Heterogeneity of MSC-Derived EVs 

A major bottleneck hampering the successful clinical application of MSC-EVs is related to their heterogeneity. First, MSC-EVs carry diverse molecules and varying amounts of bioactive components. As EVs composition is dictated by the parent cell, culturing conditions (cell source, donor variability, bioreactors vs. monolayer, presence of FBS or hPL, EVs isolation protocol) may heavily influence the biological activity of the final product. Due to an evident donor-to-donor variability of primary MSCs and its influence on the heterogeneity of MSC-EVs, the usage of immortalized MSCs for the production of EVs holds great promise in the field of overcoming various constraints linked with primary MSCs such as limited lifespan and proliferative capacity. In-process quality control that would identify changes in the EV composition and the integrity of the genome from the beginning to the end of EV production would assure the preservation of bioactive components during MSC-EVs production. Secondly, MSC-EVs comprise a heterogenous mixture of different EVs. This heterogeneity is closely related to the plethora of methods applied for their isolation/enrichment (Table 2). The composition, size, and morphology of EVs can influence their uptake, biodistribution, and functionality. Variability in surface markers expressed can also vary depending on the isolation method, culturing conditions, and donor variability, which can all influence the targeting, binding, and recognition of EVs by recipient cells and the mechanisms triggered in the target cell. 

Overcoming the heterogeneity of MSC-derived EVs is essential for improving their therapeutic efficacy, reproducibility, and clinical translation. Efforts in the field are being made to establish standardized protocols for the growth of MSCs, as well as MSC-EV isolation and characterization. 

### 5.3. MSC-EVs—Proposed Mechanisms of Action

Numerous studies have demonstrated the therapeutic effects of MSC-EVs in different disease models; nevertheless, only a limited number described their precise MoA. Here, we provide an overview of possible mechanisms of MSC-EV´s actions with an emphasis on kidney disease (Table 2). 

#### 5.3.1. Proposed MSC-EV’s Mechanisms of Action in Kidney Diseases 

A growing body of evidence suggests the significance of particular proteins in transmitting information from MSC-EVs to their target cells. Thus, human UC-MSC-EVs attenuated renal tubulointerstitial fibrosis (TIF) by delivering casein kinase 1δ (CK1δ) and E3 ubiquitin ligase (β-TRCP) to kidney fibroblasts, which promoted nuclear-cytoplasmic shuttling of Yes-associated protein (YAP) and its ubiquitination and degradation [89]. EVs from GDNF-stimulated human AD-MSCs ameliorated the loss of peritubular capillaries in the TIF model and in HUVECs through the SIRT1/eNOS signaling pathway [72]. UC-MSC-derived EVs are protected from cisplatin-induced renal oxidative stress and apoptosis by promoting proliferation through the activation of the ERK1/2 pathway [78]. There are also indications that MSC-EVs can sequester soluble molecules, hindering their biological effect. Thus, one of the highly abundant proteins on MSC-EVs, the C-C motif chemokine receptor-2 (CCR2), was able to bind to its ligand CCL2 and reduce its concentration suppressing recruitment and activation of macrophages in renal IRI in mice [55]. 

The therapeutic effect of MSC-EVs may also be mediated by the RNA. The study by Collino et al. demonstrated that miRNA from MSC-EVs might exert their effects on genes involved in fatty acid metabolism, inflammation, matrix-receptor interaction, and cell adhesion [60]. MSC-EVs were shown to transfer miR-let7c to kidney epithelial cells and downregulate the expression of TGF-βR1, attenuating kidney injury and fibrosis [61]. EVs from erythropoietin-treated MSCs transferred miR-144 to fibroblasts, thus attenuating kidney fibrosis via a decrease of tissue plasminogen activator (tPA) and MMP9 [63]. BM-MSC-EVs were also shown to protect against renal IRI by transferring miR-199a-3p to kidney cells, which downregulated semaphorin 3A expression and activated the protein kinase B (AKT) and ERK pathways [56]. Transfer of miR-30b by UC-MSC-EVs was shown to inhibit mitochondrial fission, one of the main contributors to IRI [81]. miR-146b from MSC-EVs targeted interleukin (IL)-1 receptor-associated kinase (IRAK1) leading to the inhibition of NF-κB activity and subsequent lessening of pro-inflammatory response in sepsis-associated AKI [87]. Another miRNA from MSC-EVs, miR-125b-5p, after being taken up by kidney cells markedly decreased the expression of p53, leading to the upregulated CDK1 and Cyclin B1, and modulation of Bcl-2 and Bax [88]. Furthermore, in a rat model of renal IRI, human UC-MSC-EVs alleviated kidney damage and enhanced angiogenesis via the transfer of VEGF directly to renal tubular epithelial cells. 

**Table 2:** Proposed mechanisms of MSC-EVs in kidney disease models.

MSCs Origin	MSCs Manipulation	MSCs Medium before EVs Isolation	EVs Isolation Method	Disease Model	Effects	Target Cells	Mechanisms (Molecule(s) or Signaling Pathways Identified)	Confirmation Method	Ref. No.
hUC	/	serum-free DMEM	UC	rat UUO model of RF	Amelioration of RF	/	EVs transfer CK1δ and E3 ubiquitin ligase β-TRCP promoting YAP ubiquitination and degradation	knockdown of CK1δ and β-TRCP in MSCs	[89]
hAT	transfection of GDNF in MSCs	FCS-free RPMI supplemented with 0.5% BSA	UC	mouse UUO model of RF	Amelioration of RF and peritubular capillary loss	HUVEC cells	Sirtuin 1 (SIRT1) pathway	SIRT1 knockdown	[72]
hUC	/	low glucose DMEM	UC	rat cisplatin-induced AKI	Improved renal function, morphology, and renal cell proliferation; decreased tubular apoptosis and oxidative stress	NRK-52E	p-p38 and caspase 3; PCNA and p-ERK	Inhibition of MEK1 and MEK2 with U0126	[78]
mBM	/	medium containing EVs-free serum	UC	mouse kidney IRI	Reduction of renal injury	/	CCR2 on MSC-exo binds to CCL2 lowering the recruitment of macrophages	CCR2 knockdown	[55]
BM	/	FCS-deprived RPMI with 0.5% BSA	UC	glycerol- induced AKI in severe combined IDM	Morphologic and functional recovery in AKI	/	microRNAs	Depletion of Drosha to alter miRNA expression	[60]
hBM	overexpression of miRNA-let7c	Alpha-MEM with EVs-free FBS	“ExoQuick”	mouse UUO model of RF	Attenuation of kidney injury	NRK-52E	miR-let7c attenuates TGF-β1-driven TGF-βR1 gene expression	Labeling pre-miRNA; inhibition of EVs released by GW4869	[61]
mBM	Erythropoietin treatment	RPMI with 10% FBS	UC or “PureExo exosome isolation kit”	mouse UUO model of RF	Decrease of tBM disruption	NRK-49F	miR-144 targets the tPA 3′-untranslated region and suppresses tPA and MMP9 level and activity	Depletion of miR-144	[63]
hBM	miR-199a-3p overexpression	DMEM/F12 with 10% EVs-free FBS	UC	hypoxia/reoxygenation (H/R) injury of HK-2	Alleviation of IRI, functional recovery, and histologic protection; antiapoptotic effect	HK-2	miR-199a-3p decrease Sema3A, activate protein kinase B (AKT) and extracellular-signal-regulated kinase (ERK) pathways	miR-199a-3p knockdown; Sema3A knockdown	[56]
hUC	/	FBS-depleted DMEM with 0.5% BSA	UC	rat IRI	Amelioration of acute kidney IRI; reduction of cell apoptosis	rat RTECs	miR-30b/c/d inhibits mitochondrial fission through Dynamin-related protein 1 (DRP1)	miR-30 antagomirs	[81]
hUC	/	DMEM with 10% EVs-depleted FBS	Sucrose /D2O cushion UC	sepsis— associated AKI	Alleviation of AKI and kidney tubular cells apoptosis; increased survival	HK-2	miR-146b inhibits NF-κB activity and translocation of NFκB p-P65; decrease of IRAK1	miR-146b mimics and inhibitors	[87]
hUC	/	FBS-free mix of MSCM and DMEM/F12	UC	murine IRI	Alleviation of ischemic AKI and decrease of renal tubular injury; attenuation of cell cycle arrest and apoptosis	HK-2	Homing of EVs by VLA-4 and LFA-1; miR-125b-5p suppresses p53 in TECs leading to rescue G2/M arrest through up-regulation of CDK1 and Cyclin B1; apoptosis inhibition via Bcl-2 and Bax	miR-125b-5p Ox and inhibition; Ox of p53; transfection with siICAM-1 and siVCAM-1	[88]
hBM	/	FCS-free RPMI supplemented with 0.5% of BSA	UC	rat IRI model	Reduction of renal function impairment	/	RNA cargo	RNase treatment	[54]
hUC	/	FBS-deprived DMEM, with 0.5% BSA	UC	rat model of unilateral AKI	Reversal of abnormal kidney structure and function; tubular cell dedifferentiation and growth; inhibition of apoptosis	tubular cells	Expression of HGF in tubar cells via its mRNA transfer; tubular cells media promote tubular cell dedifferentiation and migration via HGF/c-Met and Erk1/2 signaling pathways	RNase treatment; c-Met inhibitor or MEK inhibitor	[80]
hUC	/	FBS-free medium	UC	rat IRI model of AKI; hypoxia injury of RTECs	Reduction of RF and cell apoptosis; increase of proliferation, renal function, and capillary vessel density	/	VEGF delivery to rat RTECs; RNA	Anti-human VEGF antibody; RNase treatment	[84]

hUC—human umbilical cord, hAT—human adipose tissue, mBM—mouse bone marrow, hBM—human bone marrow, GDNF—Glial cell line-derived neurotrophic factor, UC—ultracentrifugation, UUO—unilateral ureteral obstruction, AKI—acute kidney disease, IRI—ischaemia-reperfusion injury; RF—renal fibrosis; NRK-52E—rat kidney tubular epithelial cells; IDM—immunodeficient mice; tBM—tubular basement membrane; NRK-49F—rat renal fibroblast cell line; HK-2—human kidney proximal tubular epithelial cell line; RTECs—kidney tubular epithelial cells; MSCM—mesenchymal stem cell medium; Ox—overexpression.

Described beneficial effects of EVs were abrogated by RNase treatment [54,80,84]. Thus, UC-MSC-EVs promoted regeneration after AKI by accelerating tubular cell dedifferentiation and growth through HGF induction and Erk1/2 signaling, while the RNase treatment abrogated all MSC-EVs effects [80]. Additionally, the effect of MSC-EVs in alleviating AKI in the IRI model, based on inhibiting apoptosis and stimulating tubular epithelial cell proliferation, was shown to be ineffective after RNase treatment [54]. However, some reports caution against miR transfer as the major road to the biological activity of EVs [41,112]. By employing sophisticated purification and stringent identification criteria, Ago proteins (that bind miR in active RISC complexes) can clearly be separated from EVs. Additionally, the probability of physiological effects of miRs may depend on the abundance of one or more mRNA targets in the recipient cell, considering that as every miR may regulate multiple target mRNAs and downregulations of one mRNA may not recapitulate total miR effects [113]. 

Thus, details on the mechanism of MSC-EVs’ interaction with target cells, signal delivery, intracellular events, and the cellular response on RNA and the protein level need more clarification. MSC-EVs undoubtedly produce beneficial effects both in in vitro and in vivo models. However, for their wide clinical use in different kidney disorders, as well as in other diseases, it is necessary to elucidate the details of their MoAs. 

#### 5.3.2. Mechanism of MSC-EVs Actions Related to Immunomodulation

It has been reported that MSC-EVs exert their immunomodulatory properties on cells of both innate and adaptive immune response [114] (Supplementary Table S1). They were shown to generally induce polarization of macrophages towards an anti-inflammatory M2 phenotype and inhibit the development of pro-inflammatory M1 macrophages. Indeed, human UC-MSC-EVs alleviated inflammation of the fallopian tubes in mice by inducing macrophage polarization from M1 to M2 type via the NF-κB signaling pathway [115]. Mouse BM-MSC-EVs attenuated myocardial injury favoring the M2 anti-inflammatory state by inhibition of the TLR4/NF-κB signaling and the activation of the PI3K/Akt pathway [116]. In sepsis-induced acute respiratory distress syndrome, mBM-MSC-EVs induced a switch towards M2 macrophages and downregulated several essential proteins of glycolysis via inhibition of HIF-1α [117]. Furthermore, MSC-EV’s capacity to shift the THP-1 cell’s inflammatory phenotype towards an anti-inflammatory profile was MyD88-dependent and involved the TLR/NF-κB signaling pathway [118]. 

Various miRNAs have been shown to play a role in MSC-EV’s MoAs related to immunomodulation. Namely, Lu et al. showed that miR-223-3p from mBM-MSC-EVs attenuated the release of LPS-induced proinflammatory cytokines from macrophages and their STAT3 expression [119], while in the mouse model of lung fibrosis, hUC-MSC-EVs transferred the same miRNA to macrophages, thus increasing the circPWWP2A and NLRP3 pro-inflammatory signaling [120]. In a mouse model of sepsis, miR-27b from mBM-MSC-EVs targeted JMJD3 and inhibited the recruitment of NF-κB to the promoter region of proinflammatory genes [121]. In a spinal cord injury model, BM-MSC-EVs exerted neuroprotective effects by targeting interferon regulatory factor 5 (IRF5) expression in macrophages via miR-125a [122]. In a mouse model of atherosclerosis, miR-21a-5p from MSC-derived exosomes promoted macrophage polarization and reduced their infiltration within the atheroma by targeting KLF6 and ERK1/2 signaling pathways [123], while miR-let7 from MSC-EVs were shown to promote M2 macrophage polarization through miR-let7/HMGA2/NF-κB pathway, and suppress macrophage infiltration via miR-let7/IGF2BP1/PTEN pathway [124]. 

Diverse proteins have also been shown to play important roles in exerting MSC-EVs effects on macrophages. Thus, STAT3 from adipose-derived MSC-EVs activated the M2 phenotype through the transactivation of arginase-1, while methallothionein-2 from hBM-MSC-EVs elicited its anti-inflammatory effects in a mouse model of inflammatory bowel disease by suppressing NF-κB activation and inhibiting the phosphorylation of IκBα [125]. 

MSC-EVs have also been shown to suppress the maturation and activation of dendritic cells (DC) [126,127,128]. However, only a few studies attempt to elucidate the mechanisms of this action. Wu et al. found that BM-MSC-EVs enhanced the miR-146a and inhibited Fas expression in immature and mature DCs while reducing IL-12 expression/production of mature DCs [129]. However, the authors did not confirm whether miR-146a from MSC-EVs was responsible for described effects. hMB-MSCs-EVs halt DC maturation, decreased expression of maturation/activation markers, and secretion of pro-inflammatory cytokines, diminishing CCR7 expression after LPS stimulation. miR-21-5p was identified as a possible candidate for the modulation of CCR7 expression and DCs migration [130]. MSC-EVs can modulate NK cells by decreasing their number in the spleen or on the site of an injury [131], as well as impairing their function, which was shown to be at least in part dependent on TGFβ signaling [132]. Studies on the influence of MSC-EVs on granulocytes demonstrate their effects on neutrophils [133,134]. MSC-EVs attenuated the complement-mediated NET formation and neutrophil IL-17 production through the CD59-dependent mechanism [135]. Furthermore, treatment of neutrophiles with hUC-MSC-EVs inhibited their activity by attenuating the respiratory burst and oxidative stress [136]. 

Several studies demonstrated the influence of MSC-EVs on mast cells and indicated possible mechanisms. Thus, MSC-EVs suppressed mast cell activation through the mechanism involving an increase of prostaglandin E2 (PGE2) production and E-prostanoid 4 (EP4) receptor in mast cells [137]. Attenuation of mast cell activation, i.e., ROS generation, degranulation, and cytokine production were accompanied by the reduction of NF-κB, p-p38, p-JNK, and p-STAT5 levels [138]. MSC-EVs were also shown to suppress the proliferation of inflammatory cells while increasing regulatory T cells through TGF-β signaling [139,140] or adenosine signaling through the A2A receptor [140,141]. The effects of MSC-EVs on B cells have also been described; however, precise mechanisms are lacking [142]. 

#### 5.3.3. Mechanism of MSC-EVs Actions Related to the Regeneration 

Apart from having immunomodulatory effects, including ones involved in the control of inflammation post-injury, MSC-EVs also have effects on the repair and remodeling phases of tissue regeneration (Supplementary Table S2). They are involved in the attenuation of apoptosis, angiogenesis, and repopulation (proliferation). Moreover, they also have a damage control role by diminishing oxidative stress. Mechanisms by which MSC-EVs exert these effects are versatile. 

MSC-EVs exert their anti-oxidative effect by transferring antioxidant glutathione peroxidase 1 (GPX1) to recipient cells [143]. They can also suppress the expression of NADPH oxidase 2 (NOX2) and thus the production of reactive oxygen species [82]. These effects can also be indirect, i.e., by influencing the anti-oxidative pathway at the level of Nrf2/antioxidant response element (ARE) activity which in turn activates the expression of antioxidant genes [83]. However, mentioned effects were not observed in all models [144], i.e., in the drug-induced, liver injury mouse model, human MSC-EVs did not mitigate hepatocyte injury via modulation of oxidative stress. 

Anti-oxidative effects of MSC-EVs are closely related to anti-apoptotic effects since oxidative stress can lead to apoptosis [145]. Thus, in the above-described example of GPX1 transfer, downregulation of apoptosis was also observed [143]. MSC-EVs can also prevent apoptosis through the activation of the Wnt/β-catenin signaling pathway [146] or by downregulation of pro-inflammatory signaling activated by IL-1β, such are Erk1/2, PI3K/Akt, p38, TAK1, and NF-κB [147]. Furthermore, MSC-EVs can cause up-regulation of anti-apoptotic genes, such as Bcl-xL [144], or modulate anti-apoptotic (Akt and GSK-3β) or proapoptotic (c-JNK) factors [148]. Several mechanisms by which MSC-EVs exert anti-apoptotic effect were connected to EVs’ transfer of RNA, i.e., miRNA which can modulate mRNAs associated with apoptosis, such as caspase-3 and -7 [149] or other types of RNA such as Y-RNA-1 [150]. Anti-apoptotic effects of MSC-EVs, exerted through downregulation of methyl CpG binding protein 2 (Mecp2) [151] and by reducing the expression of PUMA (p53 upregulated modulator of apoptosis) [152], were also associated with EVs’ transfer of miRNA (miR-22 and miR-221, respectively). Similar was observed for miR-125b-5p whose transfer via MSC-EVs was related to the suppression of pro-apoptotic genes p53 and BCL2-antagonist/killer 1 (BAK1) [153]. 

MSC-EV’s ability to promote angiogenesis often relies on shuttling pro-angiogenic transcription factors, such as VEGF, IGF-1, and bFGF [75,84]. Subsequently, the activation of the VEGF pathway can also lead to the activation of the Hippo signaling pathway [154].

MSC-EVs were often found to have significant positive or negative effects on the proliferation rates of target cells, depending on the model. Thus, in liver fibrosis, MSC-EVs decreased the proliferation of hepatic stellate cells (HSCs) by transferring miR-122 [155], while in kidney tubular cells they restored proliferation after damage caused by ATP depletion [149]. The mechanisms governing the MSC-EVs’ effects of proliferation are versatile, as they can activate Wnt/β-catenin [156]), ERK1/2 [78,157], or Akt signaling pathways [157]. They can also up-regulate proliferation proteins such are PCNA and Cyclin D1 [144,158]. In some studies, the effects of MSC-EVs on proliferation were associated with EVs’ transfer of mRNA or miRNA [76]. However, it should be noted that RNase treatment of those EVs diminished their effects, pointing out issues mentioned in Section 5.3.1. MSC-EVs can also influence the proliferation rate indirectly, by transferring mRNA for IGF-1R and thus increasing the sensitivity of recipient cells to locally produced IGF-1 [159].

The above examples of a vast variety of mechanisms of MSC-EVs’ actions point both toward the heterogeneity of EVs from different sources and conditions, as well as to the heterogenous milieus that are present in different targets (i.e., the different expression of the target molecules and/or influences of other signals on the same pathways). Obviously, MSC-EVs are able to influence different processes using different mechanisms even for the same process (i.e., inflammation or regeneration). Currently, there is a lack of studies that comprehensively elucidate the mechanisms of MSC-EVs’ actions. Most studies identified only fragments of such pathways. Given the high complexity of EVs, it is conceivable that the mechanism of MSC-EVs’ actions comprises a combination of targeting molecules and active molecules, and possibly more than one of each. Furthermore, in consideration of this plethora of information about the mechanism of MSC-EVs’ actions, different origins of MSCs (adult or neonatal, different tissues) as well as different conditions of their isolation, cultivation (influence of medium) or manipulation (knockdown, overexpression, EVs release inhibition, etc.), as reviewed in [48], should be considered as they can affect cells’ physiology and thus composition/action of EVs. Also, importantly, conditions and methods of isolation of EVs can also influence their composition and actions. Finally, the methods for confirmation of molecules identified as mechanism participants should also be considered. All this urges further efforts towards the elucidation of MSC-EVs mechanisms so we can fully exploit EVs’ versatile therapeutic potentials for kidney diseases (as reviewed in Chapter 4), as well as for other pathologies. 

### 5.4. Potency Testing

Potency is an essential parameter used to assess the therapeutic activity of a drug product. Establishing a suitable potency assay is crucial for the production of MSC and MSC-EV products. However, setting up such assays, especially for MSC-EV products, is challenging [160,161]. Ideally, potency assays should be reliable, quick, replicable, and measurable, to accurately evaluate the therapeutic product’s desired function. Furthermore, such assays should satisfy specific regulatory guidelines (https://database.ich.org/sites/default/files/Q6B_Guideline.pdf, accessed on 25 February 2023) and ideally relate to the product’s MoA (www.fda.gov/media/79856/download, accessed on 25 February 2023). 

Taking into consideration that immunomodulatory activities of MSC and MSC-EVs products are considered to be an essential part of their MoA in many disease models, they are often examined in various in vitro assays, primarily focusing on T cells or macrophages. Potency assays coupled with clinical MSC vary based on their intended clinical use but are closely related to MoA. For MSCs used in the treatment of autoimmune disease or organ transplantation, potency assays include assessment of T cell proliferation or cytokine secretion, with varying formats [162,163,164]. For MSCs used in tissue repair, tests assessing cell behavior (adhesion, proliferation, differentiation, and angiogenesis) are more appropriate [165,166,167]. However, not all of these assays measure the same immunomodulatory activity, and not all activities measured are relevant to the therapeutic effects of the cells for a specific disease [160]. Previous attempts to predict the clinical effectiveness of administered MSCs using in vitro assays have been unreliable and inconsistent. Therefore, it is important that potency assays used to predict the therapeutic function of EV-based therapeutics measure activities that are directly relevant to the EV products’ MoA for a specific disease, which may be a compendium of different individual activities or MoA attributes, respectively [168]. Thus, appropriate potency testing may require an assay matrix to be established [168].

There are several aspects that have to be taken into account when designing the right potency assay for kidney diseases. The design of the potency assays for MSC-EV therapies in kidney disease will greatly depend on the pathophysiological stage of the disease, whether it is acute or chronic, and the type of underlying condition, either inflammatory or fibrotic. Thus, when designing the potency assay for the MSC-EV product that will be used for the AKI, the accent should be placed on immunomodulatory, anti-inflammatory, and renoprotective effects. Conversely, potency assays measuring the extent of ECM in kidney fibrosis may be more advantageous for MSC-EV products that will be used in the later stages of kidney disease. In addition, whenever we encounter difficulties for routine tests (due to high variability or time consumption), it is preferable to utilize surrogate measurements (indirect measures) that have been previously shown to correlate with functional assays, such as specific gene expression or secretion factors. Thus, the effect of MSC-EVs on inflammatory response in AKI can be assessed in vitro by measuring the secretion of pro- and anti-inflammatory factors, such as IL-6, TNF-α, and IL-10, while the effect of MSC-EV product on renoprotection and tubular renal senescence could be measured by assessing the levels of renal αKlotho, known antiaging and renoprotective protein.

Even though the current pre- and clinical data imply a huge potential for MSC-EV products, there are some caveats inherited from the MSC field [160]. It is obvious that MSCs are a very heterogenous cell entity [161,169,170,171] and that not all MSC products succeeded in clinical trials [172,173,174]. Namely, a comparison of therapeutic activities of independent MSC-EV preparations all manufactured with the same standardized procedure, demonstrated differences in the potency of different MSC-EV batches in modulating disease symptoms in various animal models, specifically in mouse models of ischemic stroke, Niemann-Pick type C disease and aGvHD [175,176,177]. Thus, it is an important task in the field, now, to set up assays that appropriately evaluate the therapeutic activities of MSC-EV products, allowing discrimination between potent and non-potent products before applying them to larger patient cohorts in clinical trials [160,168,178].

## 6. Perspectives of MSC-EV Use in the Treatment of Kidney Disease

While incidences of kidney disease are rising worldwide, we still lack effective treatments to repair damaged kidney tissue and/or prevent disease progression. The use of MSC technology, already proven to be promising in the treatment of a variety of diseases, emerged as such a strategy. This especially refers to their EVs, which offer important advantages to cellular therapy, modulating vital processes important for kidney physiology such as immune response, tissue repair, angiogenesis, oxidative stress, and inhibition of cell death.

The beneficial and renoprotective effects of MSC-EVs in kidney pathologies have been well documented in a large number of different preclinical models. However, their use in clinical trials is clearly very limited. One possible explanation is that there is still much that is not yet known about the mechanisms of action of MSC-EV products, their safety, efficacy, and optimal dosing regimens, as well as the lack of suitable potency assays. 

In order to make a significant step toward the clinical application of MSC-EVs, we must ensure that crucial obstacles are overcome. This involves standardization of methods for MSC-EVs isolation and characterization. Different aspects of quality control such as quantity, purity, sterility, and endotoxin should not be disregarded. In parallel, we need to clarify the sophisticated mechanisms of MSC-EVs’ action and design appropriate potency assays that will precisely assess the therapeutic activity of MSC-EV products in a specific kidney disease. In this regard, depending on the pathophysiological stage of kidney disease, assessment of the effect of MSC-EV products on kidney tubular or glomerular inflammation, apoptosis, or senescence could be considered as one of the potential in vitro potency assays. Furthermore, designing the potency assay that will work on a kidney-on-a-chip in vitro model would surpass the usual limitations of a conventional 2D-cell culture model and allow the study of the MSC-EVs´ dynamic within the nephron structure, which is very important taking into consideration the complex kidney structure.

Due to the considerable complexity of the kidney structure and the multifaceted pathogenesis of kidney diseases, kidney-targeted drug delivery is a difficult task as there are currently very limited strategies available to target the right kidney cell and tackle the precise signaling pathway. Hence, we need to work on enhancing the efficacy of MSC-EV-based renal therapy. Increasing the therapeutic efficacy of MSC-EVs, by presenting different targeting moieties such as antibodies, peptides, and small molecules on their surface would lead to better homing of MSC-EVs towards different kidney cells and a decrease in target effects. 

Despite challenges in MSC-EV production and characterization, the potential of MSC-EV-based therapeutics as a novel type of nanomedicine for kidney disease is promising. As our comprehension of kidney disease continues to thrive, the development of MSC-EV products will also continue to evolve, paving the way for an effective treatment option for patients with kidney disease. 

## Data Availability

Not applicable.

## Supplementary Materials

The following are available online at https://www.mdpi.com/article/10.3390/pharmaceutics15071911/s1, Table S1: Proposed mechanisms of MSC-EVs related to immunological effects, Table S2: Proposed mechanisms of MSC-EVs related to regenerative effects.
