# Supplementary material for: Clinical Prospect of Mesenchymal Stromal/Stem Cell-Derived Extracellular Vesicles in Kidney Disease: Challenges and the Way Forward
Source: Pharmaceutics. 2023 Jul 9;15(7):1911. doi: 10.3390/pharmaceutics15071911 (PMC10384614; doi:10.3390/pharmaceutics15071911)
Supplement: Supplementary file 1 [file pharmaceutics-15-01911-s001.zip › pharmaceutics-2445894-supplementary.pdf]

Supplementary Table S1. Proposed mechanisms of MSC-EVs related to immunological effects

MSCs origin	MSCs manipulation	MSCs medium before EVs isolation	EVs isolation method	Disease model	Effects	Target cells	Mechanism (molecule(s) or signaling pathways identified)	Confirmation method	Ref. No.
hUC	/	serum-free DMEM/F12	UC	murine chronic salpingitis	Improvement of the local inflammatory microenvironment of fallopian tubes and inhibition of tube inflammation; alleviation of tubal factor infertility	RAW 264.7	Macrophage polarization from M1 to M2 via NF-κB signaling pathway, lower expression of p65 and TLR4	/	[1]
mBM	/	medium containing 5% exosome-depleted FBS	UC	murine myocardial IRI	Polarization to M2 macrophages; reduction of infarct size and alleviation of inflammation level in heart and serum	RAW264.7	miR-182 mediator of macrophage polarization and TLR4 as a downstream target	systemic depletion of macrophages; diminishing miR-182; knockdown of TLR4	[2]
mBM	/	DMEM/F12 with 10% EVs-depleted FBS	UC	LPS-induced ARDS	Inhibition of M1 and promotion of M2 polarization in vitro; amelioration of LPS-induced inflammation and lung damage	MH-S	Inhibition of cellular glycolysis via HIF-1a inhibition	HIF-1a siRNA	[3]
hESC	/	/	TFF + HPLC + UF	mouse skin graft GVHD	Activation of APCs; induction of anti-inflammatory M2; elicitation of Treg; enhancement of allogeneic skin graft	HEK-Blue-hTLR4 and hTLR2; THP1-Xblue; THP1 XBlue-defMYD; monocytes, splenocytes	Activation of APCs via MyD88-dependent translocation of NFκB through TLR4; M2 induction through MyD88 only in I phase; Treg induction via M2-like monocytes, MYD88-dependent	use of MyD88-deficient cells	[4]

MSCs origin	MSCs manipulation	MSCs medium before EVs isolation	EVs isolation method	Disease model	Effects	Target cells	Mechanism (molecule(s) or signaling pathways identified)	Confirmation method	Ref. No.
BM	transfection of miR-223-3p	a-MEM medium	UC	autoimmune hepatitis	Decrease of inflammatory cytokines from liver and macrophages; lower inflammatory responses in the liver; elevation of the Treg/Th17 ratio	RAW264.7	miRNA-223-3p-mediated negative regulation of STAT3, IL-1β and IL-6 expressions	miR-223 knockdown	[5]
hUC	/	/	"ExoQuick"	SLF	Attenuation of respiratory system function, NLRP3 activation, and fibrosis	RAW264.7	EVs affect circPWWP2A/ miR-223–3p /NLRP3 regulatory pathway	siRNAs of NLRP3 siRNA of circPWWP2A; miR-223–3p mimics	[6]
mBM	/	FBS-free RPMI-1640	UC	mouse sepsis	Inhibition of CLP-induced sepsis	BM-derived macrophages	miRNA-27b targets JMJD3 and downregulates NF-κB	miR-27b knockdown; miR-27b-mimic	[7]
rat BM	/	serum-free stem cell medium	sucrose/D2O cushion UC	rat SCI	Amelioration of SCI symptoms; promotion of M2 polarization	RAW264.7	miR-125a from EVs inhibits IRF5 expression	overexpression of IRF5; miR-125a knockdown	[8]
mBM	/	RPMI 1640 medium	"Total Exosome Isolation Reagent Kit"	atherosclerosis	Reduction of plaque area and macrophage infiltration; M2 polarization	RAW264.7	miR-21a-5p targets KLF6 and ERK2 suppressing KLF6 and ERK1/2 -> M2 polarization, inhibition of migration	miR-21a-5p overexpression in RAW264.7 cells	[9]
mBM	/	medium with EVs-free serum	TFF+HPLC+UF	atherosclerosis	Decrease of atherosclerotic plaque; reduction of macrophage infiltration; M2 polarization	U-937	miR-let7 induces M2 polarization via miR-let7/HMGA2/NF-kB and suppresses macrophage infiltration via miR-let7/IGF2BP1/PTEN	miR-let7 mimics and inhibitors in U937 cells	[10]
hBM	/	α-MEM with 10% exosome-depleted FBS	UC	IBD models of experimental colitis	Mitigation of colitis; downregulation of inflammatory responses; maintenance of intestinal barrier integrity M2 polarization and IL-10 release	Monocyte-derived macrophages	MT-2 from EVs is necessary for uptake into macrophages; MT-2 inhibits inflammatory response via attenuation of NF-κB, i.e., by modulating IκBα expression via interacting with MZF1	Depletion of colonic macrophage; Neutralization of IL-10; MT-2 knockdown	[11]

MSCs origin	MSCs manipulation	MSCs medium before EVs isolation	EVs isolation method	Disease model	Effects	Target cells	Mechanism (molecule(s) or signaling pathways identified)	Confirmation method	Ref. No.
mBM	/	DMEM-LG medium	"ExoQuick"	mice kidney transplant model	Prolong the allogenic kidney graft survival	DCs	Inhibiting DCs maturity by increasing miR-146a in DCs; reduction of IL-12 mRNA expression and IL-12 production of mDCs	miR-146a silencing	[12]
hBM	/	basal medium supplemented with 10% human platelets lysate	UC	/	Impairment of antigen uptake by immature DCs; impairment of DC migration	DCs	Impairment of DC migration via suppression of CCR7; miRNA-21-5p partially mimics the function of MSC-EV treatment on DCs	miR-21-5p overexpression in DCs	[13]
hUC	/	DMEM with 0.5% BSA	UC	rat model of renal IRI	Suppression of IRI-induced up-regulation of NK cells in spleen and ischemic kidney; protective role after the removal of spleen in IRI rats	HUVECs; NRK-52E	RNA involvement; downregulation of CX3CL1 and TLR-2	Depletion of NK cells; RNA-specific fluorescent dye (SYTO)	[14]
hFL	/	exosome-depleted FBS a-MEM complete medium	UC	/	Impairment of NK cell function	human NK cells	Activation of TGFβ/Smad pathway in NK cells by EVs, via TGFb on their surface	Detection of pSmad2/3; anti-TGFβ neutralizing antibody	[15]
hESCs	/	supplemented DMEM	TFF + UF	/	Inhibition of complement-induced neutrophil activation	neutrophils	EVs inhibit complement-induced neutrophil activation through a CD59-dependent mechanism	anti-CD59 antibody abrogated the inhibitory effects of EVs	[16]
hBM	/	serum-deprived medium	UC	mouse intra-cranial aneurysm model	Reduction of aneurysmal rupture rate; suppression of mast cells activation	primary cultures of murine mast cells; LAD2	EVs cause the upregulation of PGE2 production via up-regulation of mRNA expression of COX2 and upregulation of EP4 receptor expression	COX2 inhibitor; EP4 antagonist	[17]

MSCs origin	MSCs manipulation	MSCs medium before EVs isolation	EVs isolation method	Disease model	Effects	Target cells	Mechanism (molecule(s) or signaling pathways identified)	Confirmation method	Ref. No.
hUC	/	α-MEM containing 5% human platelet lysate	TFF + diafiltration	/	Attenuation of induced ROS generation, degranulation, and production of proinflammatory cytokines; inhibition of inflammatory and allergic reactions	KU812 cells	Suppression of NF-kB and MAPK	/	[18]
endometrium (men-strual blood)	/	DMEM containing 1% insulin-transferrin-selenium	UC	/	T cell activation	PBLs	EVs inhibitory effect against CD4+ T cell activation is mediated via TGFβ signaling	Anti-TGFβ neutralizing antibodies	[19]
canine-UC	/	serum-free supple-mented DMEM	UC	/	Inhibition of CD4+ T cell proliferation	T cells	TGFβ and adenosine signaling	TGFβRI antagonist; neutralizing antibodies to TGFβ; A2A adenosine receptor block	[20]
hUC; BM	/	serum-free medium	UC	/	Suppressed proliferation of T cells	T cells	CD39-expressing T cells produce AMP from ATP and MSCs produce adenosine from AMP via CD73, causing immunosuppression of T cells	/	[21]
hBM	/	DMEM supplemented with EVs-depleted FBS	UC	/	EVs decrease the proliferation of activated PBMCs or isolated T and B cells;<br>inhibited the production of IgM by B cells	PBMCs; isolated B and T lymphocyte	Increase of CXCL8 and MZB1 in B cells upon EVs treatment	/	[22]

Supplementary Table S2. Proposed mechanisms of MSC-EVs related to regenerative effects

Origin of MSC	MSCs manipulation	MSCs medium before EVs isolation	EVs isolation method	Disease model	Effects	Target cells	Mechanism (molecule(s) or signaling pathways identified)	Confirmation method	Ref. No.
hUC	/	FBS-deprived DMEM with 0.5% BSA	UC	renal IRI in rats	Decline of ROS, oxidative stress, and fibrosis; ameliorated renal function; reduced apoptosis and enhanced proliferation	NRK-52E; HUVECs	NOX2 downregulation	NOX2 downregulation both *in vivo* and *in vitro*	[23]
hUC	/	FBS-deprived DMEM with 0.5% BSA	UC	renal IRI in rats	Alleviation of renal tissue injury and apoptosis	NRK-52E	Activation of Nrf2/ARE, up-regulation of HO-1	comparison to non-MSC EVs	[24]
hESC-derived HuES9.E1 MSCs	/	serum-free culture medium	TFF + UF + HPLC	liver injury	Increase in hepatocyte proliferation, viability, and survival rate; induction of quiescent hepatocytes (G0) to re-enter the cell cycle (G1)	3 hepato-cytes cell lines: TAMH, THLE-2, HuH-7	Induction of regenerative genes (NF-κB, cyclin D1, and cyclin E); protection from apoptosis by decreasing caspase 3/7 level while upregulating Bcl-xL; induction of transcription factors expression during the G1 phase	dose dependency	[25]
rat BM	/	EVs-depleted FBS-containing medium	UC	rat severe SCI	Improvement of locomotor functional recovery; inhibition of neuronal apoptosis	neuron cells from the spinal cord	Wnt/β-catenin	Wnt/β-catenin signaling pathway inhibitor	[26]
hBM	/	α-MEM medium with EVs depleted-FCS	UC	osteoarthritis	Promotion of proliferation, migration, and reduction of apoptosis; alleviation of IL-1β-induced catabolic effects on chondrocytes from osteoarthritis	chondrocytes from osteoarthritis patients	Downregulation of Erk1/2, PI3K/Akt, p38, TAK1, and NF-κB signaling pathways	/	[27]
hESC-derived HuES9.E1 MSCs	/	supplemented DMEM	TFF+HPLC+UF	IRI model of MCI in mice	Reduction of IRI; improvement of cardiac performance; reduction of oxidative stress and inflammation	/	Activation of PI3K/Akt pathway	/	[28]

Origin of MSC	MSCs manipulation	MSCs medium before EVs isolation	EVs isolation method	Disease model	Effects	Target cells	Mechanism (molecule(s) or signaling pathways identified)	Confirmation method	Ref. No.
hBM	/	RPMI containing 0.5% BSA	UC	*in vitro* IRI of renal cells; ATP depletion injury	Reduction of transepithelial resistance loss	HK-2	Uptake via CD229 and CD44; delivery and upregulation of miRNA; reversal of miRNA changes in target cell; downregulation of coding-mRNAs associated with apoptosis, cytoskeleton reorganization, and hypoxia, such as CASP3 and 7, SHC1 and SMAD4	Blocking EVs uptake by antibody to CD29 or hyaluronic acid to block CD44; transcription inhibition by actinomycin D	[29]
mBM hBM	/	Vesicle-depleted medium	UC	lethal murine model of hepatic failure	Reduction of inflammation and hepatic injury; modulation of cytokine expression; survival increase	human hepatocytes	lncRNA Y-RNA-1 from EVs mediates the reduction of apoptosis in hepatocytes	siRNA-mediated knockdown of Y-RNA-1 in hBM-MSC	[30]
mBM	ISCP	serum-free medium	UC, ExoQuick	mouse MCI	Reduction of cardiomyocyte apoptosis; amelioration of fibrosis post-MCI	neonatal cardiomyocytes	miR-22 carried by EV target Mecp2 (methyl CpG binding protein 2) which is upregulated in infarcted hearts	miR-22 mimic; miR-22 inhibitor; knockdown of Mecp2 by siRNAs	[31]
rat BM	GATA-4 overexpression	serum- and antibiotic-free medium	ExoQuick	cardiomyocytes ischemic injury, *in vitro* model of MCI	Reduction of apoptosis; cardioprotection	neonatal rat ventricle cardiomyocytes	GATA-4 increases miR-221 in MSC; EVs transport miR-221 to cardiomyocytes reducing the expression of PUMA, i.e., reducing apoptosis	Overexpression of miR 221 in MSCs; co-transfection of lenti-miR-221 vector and full-length 3′ UTR sequence of PUMA	[32]
mBM	hypoxia-conditioning	EVs depleted α-MEM	UC	mouse MCI model	Improvement of cardiac function; attenuation of apoptosis	H9C2 rat cardiomyoblast	Improved targeted delivery of EVs by conjugating them with a “CSTSMLKAC” peptide; anti-apoptotic effect of miR-125b contained in EVs	knockdown of miR-125b-5p	[33]

Origin of MSC	MSCs manipulation	MSCs medium before EVs isolation	EVs isolation method	Disease model	Effects	Target cells	Mechanism (molecule(s) or signaling pathways identified)	Confirmation method	Ref. No.
rat BM	/	EVs-depleted FBS-containing medium	UF + sucrose /D2O cushion-UC	rat rotator cuff recon-struction	Promotion of proliferation, migration, and angiogenic tube formation in HUVECs; promotion of angiogenesis around the tendon-bone interface of the rotator cuff; lower inflammation by the inhibition of M1 macrophage polarization	U937 cells; HUVECs	Activation of the angiogenic signaling pathway, i.e., VEGFR phosphorylation which inhibits phosphorylation of LATS1/2 and YAP1	VEGFR inhibitor (Nintedanib)	[34]
hAT	LV-mediated transfer of pre-miR-122	MesenPRO RS™ medium	"ExoQuick"	liver fibrosis	Suppression of the proliferation and collagen maturation in LX-2 cells; alleviation of collagen deposition	LX-2	miR-122I targets IGF1R, CCNG1 and P4HA1	/	[35]
hUC	/	serum-free DMEM	UF + sucrose/D2O cushion-UC	rat BIS	Promotion of cell proliferation and re-epithelialization; inhibition of apoptosis	HLF; Keratinocytes, HaCAT; Dermal fibroblasts	Parallel activation of Wnt4/β-catenin and AKT signaling	knockdown of Wnt4; inhibition of AKT	[36]
h	/	/	sequential TFF	rat periodontal defect	Promotion of regeneration of critical-size periodontal defects; proliferation and cell migration increase	periodontal ligament cells	AR-mediated activation of AKT and ERK signaling pathways	wortmannin (inhibitor of PI3K/AKT pathway); U0126 (inhibitor of MAPK/ERK pathway)	[37]
h gingiva	/	a-MEM with 1% EVs depleted FBS	UF + 'ExoQuick'	crush injury of mice sciatic nerve	Promotion of axonal repair and functional recovery of the crush-injured mice's sciatic nerves; promotion of proliferation and migration of Schwann cells	rat Schwann cell line RT4-D6P2T; primary rat Schwann cells	Upregulation of the expression of genes, driving the dedifferentiation and activation of the repair phenotype of Schwann cells: c-JUN and Notch1	GW4869, an exosome/EVs inhibitor	[38]

Origin of MSC	MSCs manipulation	MSCs medium before EVs isolation	EVs isolation method	Disease model	Effects	Target cells	Mechanism (molecule(s) or signaling pathways identified)	Confirmation method	Ref. No.
hBM; mBM	/	medium containing EVs-free platelet lysate	UC	/	Regeneration of damaged RPTECs; increase of RPTEC´s proliferation	hDF human, mouse RPTECs; HK2	EVs transfer IGF-1R mRNA	Silencing of IGF-1 receptor	[39]

hUC – human umbilical cord, hAT – human adipose tissue, mBM – mouse bone marrow, hBM – human bone marrow, hFL - human fetal liver UC – ultracentrifugation, TFF – tangential flow filtration; HPLC – high performance liquid chromatography; UUO – unilateral ureteral obstruction, AKI – acute kidney disease, IRI - ischemia-reperfusion injury; RAW 264.7 (mouse macrophage cell line); U-937 (human monocyte cell line); murine alveolar macrophage cell line MH-S; HIF-1a - hypoxia-inducible factor 1 alpha; TLR4 - toll-like receptor 4; SCI - model of spinal cord injury; MZF1 - myeloid zinc finger 1; LAD2 - human mast cell line; PGE2 - prostaglandin E2; EP4 - E-prostanoid 4; HMC-1 - human mast cell line; KU812 - immature human basophilic leukocyte; PBLs - peripheral blood lymphocytes; PBMCs - peripheral blood mononuclear cells; MCI - myocardial infarction; HK-2 (renal proximal tubular epithelial cells); ISCP - ischemic preconditioning; PUMA - p53 upregulated modulator of apoptosis; LX-2 - hepatic stellate cells line; LV – lentivirus; BIS - deep second-degree burn injury skin wound model; HLF - human lung fibroblasts; HaCaT - spontaneously immortalized keratinocyte cell line from the adult human skin; AR - adenosine receptor; RPTECs – renal proximal tubular epithelial cells; hDF – human dermal fibroblasts; MT2 - Metallothionein 2; hESC - huES9.E1 cell line; SLF - model of silicosis lung fibrosis; NK - natural killer cells.

References

1. Zhang, C.; Liao, W.; Li, W.; Li, M.; Xu, X.; Sun, H.; Xue, Y.; Liu, L.; Qiu, J.; Zhang, X.; et al. Human umbilical cord mesenchymal stem cells derived extracellular vesicles alleviate salpingitis by promoting M1-to-M2 transformation. *Front Physiol* **2023**, *14*, 1131701, doi:10.3389/fphys.2023.1131701.
2. Zhao, J.; Li, X.; Hu, J.; Chen, F.; Qiao, S.; Sun, X.; Gao, L.; Xie, J.; Xu, B. Mesenchymal stromal cell-derived exosomes attenuate myocardial ischaemia-reperfusion injury through miR-182-regulated macrophage polarization. *Cardiovasc Res* **2019**, *115*, 1205-1216, doi:10.1093/cvr/cvz040.
3. Deng, H.; Wu, L.; Liu, M.; Zhu, L.; Chen, Y.; Zhou, H.; Shi, X.; Wei, J.; Zheng, L.; Hu, X.; et al. Bone Marrow Mesenchymal Stem Cell-Derived Exosomes Attenuate LPS-Induced ARDS by Modulating Macrophage Polarization Through Inhibiting Glycolysis in Macrophages. *Shock* **2020**, *54*, 828-843, doi:10.1097/SHK.0000000000001549.
4. Zhang, B.; Yin, Y.; Lai, R.C.; Tan, S.S.; Choo, A.B.; Lim, S.K. Mesenchymal stem cells secrete immunologically active exosomes. *Stem Cells Dev* **2014**, *23*, 1233-1244, doi:10.1089/scd.2013.0479.
5. Lu, F.B.; Chen, D.Z.; Chen, L.; Hu, E.D.; Wu, J.L.; Li, H.; Gong, Y.W.; Lin, Z.; Wang, X.D.; Li, J.; et al. Attenuation of Experimental Autoimmune Hepatitis in Mice with Bone Mesenchymal Stem Cell-Derived Exosomes Carrying MicroRNA-223-3p. *Mol Cells* **2019**, *42*, 906-918, doi:10.14348/molcells.2019.2283.
6. Hou, L.; Zhu, Z.; Jiang, F.; Zhao, J.; Jia, Q.; Jiang, Q.; Wang, H.; Xue, W.; Wang, Y.; Tian, L. Human umbilical cord mesenchymal stem cell-derived extracellular vesicles alleviated silica induced lung inflammation and fibrosis in mice via circPWWP2A/miR-223-3p/NLRP3 axis. *Ecotoxicol Environ Saf* **2023**, *251*, 114537, doi:10.1016/j.ecoenv.2023.114537.
7. Sun, J.; Sun, X.; Chen, J.; Liao, X.; He, Y.; Wang, J.; Chen, R.; Hu, S.; Qiu, C. microRNA-27b shuttled by mesenchymal stem cell-derived exosomes prevents sepsis by targeting JMJD3 and downregulating NF-κB signaling pathway. *Stem Cell Res Ther* **2021**, *12*, 14, doi:10.1186/s13287-020-02068-w.
8. Chang, Q.; Hao, Y.; Wang, Y.; Zhou, Y.; Zhuo, H.; Zhao, G. Bone marrow mesenchymal stem cell-derived exosomal microRNA-125a promotes M2 macrophage polarization in spinal cord injury by downregulating IRF5. *Brain Res Bull* **2021**, *170*, 199-210, doi:10.1016/j.brainresbull.2021.02.015.
9. Ma, J.; Chen, L.; Zhu, X.; Li, Q.; Hu, L.; Li, H. Mesenchymal stem cell-derived exosomal miR-21a-5p promotes M2 macrophage polarization and reduces macrophage infiltration to attenuate atherosclerosis. *Acta Biochim Biophys Sin (Shanghai)* **2021**, *53*, 1227-1236, doi:10.1093/abbs/gmab102.
10. Li, J.; Xue, H.; Li, T.; Chu, X.; Xin, D.; Xiong, Y.; Qiu, W.; Gao, X.; Qian, M.; Xu, J.; et al. Exosomes derived from mesenchymal stem cells attenuate the progression of atherosclerosis in ApoE. *Biochem Biophys Res Commun* **2019**, *510*, 565-572, doi:10.1016/j.bbrc.2019.02.005.
11. Liu, H.; Liang, Z.; Wang, F.; Zhou, C.; Zheng, X.; Hu, T.; He, X.; Wu, X.; Lan, P. Exosomes from mesenchymal stromal cells reduce murine colonic inflammation via a macrophage-dependent mechanism. *JCI Insight* **2019**, *4*, doi:10.1172/jci.insight.131273.
12. Wu, X.Q.; Yan, T.Z.; Wang, Z.W.; Wu, X.; Cao, G.H.; Zhang, C. BM-MSCs-derived microvesicles promote allogeneic kidney graft survival through enhancing micro-146a expression of dendritic cells. *Immunol Lett* **2017**, *191*, 55-62, doi:10.1016/j.imlet.2017.09.010.
13. Reis, M.; Mavin, E.; Nicholson, L.; Green, K.; Dickinson, A.M.; Wang, X.N. Mesenchymal Stromal Cell-Derived Extracellular Vesicles Attenuate Dendritic Cell Maturation and Function. *Front Immunol* **2018**, *9*, 2538, doi:10.3389/fimmu.2018.02538.
14. Zou, X.; Gu, D.; Zhang, G.; Zhong, L.; Cheng, Z.; Liu, G.; Zhu, Y. NK Cell Regulatory Property is Involved in the Protective Role of MSC-Derived Extracellular Vesicles in Renal Ischemic Reperfusion Injury. *Hum Gene Ther* **2016**, *27*, 926-935, doi:10.1089/hum.2016.057.
15. Fan, Y.; Herr, F.; Vernochet, A.; Mennesson, B.; Oberlin, E.; Durrbach, A. Human Fetal Liver Mesenchymal Stem Cell-Derived Exosomes Impair Natural Killer Cell Function. *Stem Cells Dev* **2019**, *28*, 44-55, doi:10.1089/scd.2018.0015.

16. Loh, J.T.; Zhang, B.; Teo, J.K.H.; Lai, R.C.; Choo, A.B.H.; Lam, K.P.; Lim, S.K. Mechanism for the attenuation of neutrophil and complement hyperactivity by MSC exosomes. *Cytotherapy* **2022**, *24*, 711-719, doi:10.1016/j.jcyt.2021.12.003.
17. Liu, J.; Kuwabara, A.; Kamio, Y.; Hu, S.; Park, J.; Hashimoto, T.; Lee, J.W. Human Mesenchymal Stem Cell-Derived Microvesicles Prevent the Rupture of Intracranial Aneurysm in Part by Suppression of Mast Cell Activation via a PGE2-Dependent Mechanism. *Stem Cells* **2016**, *34*, 2943-2955, doi:10.1002/stem.2448.
18. Lin, T.Y.; Chang, T.M.; Huang, H.C. Extracellular Vesicles Derived from Human Umbilical Cord Mesenchymal Stem Cells Attenuate Mast Cell Activation. *Antioxidants (Basel)* **2022**, *11*, doi:10.3390/antiox11112279.
19. Álvarez, V.; Sánchez-Margallo, F.M.; Macías-García, B.; Gómez-Serrano, M.; Jorge, I.; Vázquez, J.; Blázquez, R.; Casado, J.G. The immunomodulatory activity of extracellular vesicles derived from endometrial mesenchymal stem cells on CD4+ T cells is partially mediated by TGFbeta. *J Tissue Eng Regen Med* **2018**, *12*, 2088-2098, doi:10.1002/term.2743.
20. Crain, S.K.; Robinson, S.R.; Thane, K.E.; Davis, A.M.; Meola, D.M.; Barton, B.A.; Yang, V.K.; Hoffman, A.M. Extracellular Vesicles from Wharton's Jelly Mesenchymal Stem Cells Suppress CD4 Expressing T Cells Through Transforming Growth Factor Beta and Adenosine Signaling in a Canine Model. *Stem Cells Dev* **2019**, *28*, 212-226, doi:10.1089/scd.2018.0097.
21. Kerkelä, E.; Laitinen, A.; Räbinä, J.; Valkonen, S.; Takatalo, M.; Larjo, A.; Veijola, J.; Lampinen, M.; Siljander, P.; Lehenkari, P.; et al. Adenosinergic Immunosuppression by Human Mesenchymal Stromal Cells Requires Co-Operation with T cells. *Stem Cells* **2016**, *34*, 781-790, doi:10.1002/stem.2280.
22. Khare, D.; Or, R.; Resnick, I.; Barkatz, C.; Almogi-Hazan, O.; Avni, B. Mesenchymal Stromal Cell-Derived Exosomes Affect mRNA Expression and Function of B-Lymphocytes. *Front Immunol* **2018**, *9*, 3053, doi:10.3389/fimmu.2018.03053.
23. Zhang, G.; Zou, X.; Miao, S.; Chen, J.; Du, T.; Zhong, L.; Ju, G.; Liu, G.; Zhu, Y. The anti-oxidative role of micro-vesicles derived from human Wharton-Jelly mesenchymal stromal cells through NOX2/gp91(phox) suppression in alleviating renal ischemia-reperfusion injury in rats. *PLoS One* **2014**, *9*, e92129, doi:10.1371/journal.pone.0092129.
24. Zhang, G.; Zou, X.; Huang, Y.; Wang, F.; Miao, S.; Liu, G.; Chen, M.; Zhu, Y. Mesenchymal Stromal Cell-Derived Extracellular Vesicles Protect Against Acute Kidney Injury Through Anti-Oxidation by Enhancing Nrf2/ARE Activation in Rats. *Kidney Blood Press Res* **2016**, *41*, 119-128, doi:10.1159/000443413.
25. Tan, C.Y.; Lai, R.C.; Wong, W.; Dan, Y.Y.; Lim, S.K.; Ho, H.K. Mesenchymal stem cell-derived exosomes promote hepatic regeneration in drug-induced liver injury models. *Stem Cell Res Ther* **2014**, *5*, 76, doi:10.1186/scrt465.
26. Li, C.; Jiao, G.; Wu, W.; Wang, H.; Ren, S.; Zhang, L.; Zhou, H.; Liu, H.; Chen, Y. Exosomes from Bone Marrow Mesenchymal Stem Cells Inhibit Neuronal Apoptosis and Promote Motor Function Recovery via the Wnt/β-catenin Signaling Pathway. *Cell Transplant* **2019**, *28*, 1373-1383, doi:10.1177/0963689719870999.
27. Li, S.; Stöckl, S.; Lukas, C.; Götz, J.; Herrmann, M.; Federlin, M.; Grässel, S. hBMSC-Derived Extracellular Vesicles Attenuate IL-1β-Induced Catabolic Effects on OA-Chondrocytes by Regulating Pro-inflammatory Signaling Pathways. *Front Bioeng Biotechnol* **2020**, *8*, 603598, doi:10.3389/fbioe.2020.603598.
28. Arslan, F.; Lai, R.C.; Smeets, M.B.; Akeroyd, L.; Choo, A.; Aguor, E.N.; Timmers, L.; van Rijen, H.V.; Doevendans, P.A.; Pasterkamp, G.; et al. Mesenchymal stem cell-derived exosomes increase ATP levels, decrease oxidative stress and activate PI3K/Akt pathway to enhance myocardial viability and prevent adverse remodeling after myocardial ischemia/reperfusion injury. *Stem Cell Res* **2013**, *10*, 301-312, doi:10.1016/j.scr.2013.01.002.

29. Lindoso, R.S.; Collino, F.; Bruno, S.; Araujo, D.S.; Sant'Anna, J.F.; Tetta, C.; Provero, P.; Quesenberry, P.J.; Vieyra, A.; Einicker-Lamas, M.; et al. Extracellular vesicles released from mesenchymal stromal cells modulate miRNA in renal tubular cells and inhibit ATP depletion injury. *Stem Cells Dev* **2014**, *23*, 1809-1819, doi:10.1089/scd.2013.0618.
30. Haga, H.; Yan, I.K.; Takahashi, K.; Matsuda, A.; Patel, T. Extracellular Vesicles from Bone Marrow-Derived Mesenchymal Stem Cells Improve Survival from Lethal Hepatic Failure in Mice. *Stem Cells Transl Med* **2017**, *6*, 1262-1272, doi:10.1002/sctm.16-0226.
31. Feng, Y.; Huang, W.; Wani, M.; Yu, X.; Ashraf, M. Ischemic preconditioning potentiates the protective effect of stem cells through secretion of exosomes by targeting Mecp2 via miR-22. *PLoS One* **2014**, *9*, e88685, doi:10.1371/journal.pone.0088685.
32. Yu, B.; Gong, M.; Wang, Y.; Millard, R.W.; Pasha, Z.; Yang, Y.; Ashraf, M.; Xu, M. Cardiomyocyte protection by GATA-4 gene engineered mesenchymal stem cells is partially mediated by translocation of miR-221 in microvesicles. *PLoS One* **2013**, *8*, e73304, doi:10.1371/journal.pone.0073304.
33. Zhu, L.P.; Tian, T.; Wang, J.Y.; He, J.N.; Chen, T.; Pan, M.; Xu, L.; Zhang, H.X.; Qiu, X.T.; Li, C.C.; et al. Hypoxia-elicited mesenchymal stem cell-derived exosomes facilitates cardiac repair through miR-125b-mediated prevention of cell death in myocardial infarction. *Theranostics* **2018**, *8*, 6163-6177, doi:10.7150/thno.28021.
34. Huang, Y.; He, B.; Wang, L.; Yuan, B.; Shu, H.; Zhang, F.; Sun, L. Bone marrow mesenchymal stem cell-derived exosomes promote rotator cuff tendon-bone healing by promoting angiogenesis and regulating M1 macrophages in rats. *Stem Cell Res Ther* **2020**, *11*, 496, doi:10.1186/s13287-020-02005-x.
35. Lou, G.; Yang, Y.; Liu, F.; Ye, B.; Chen, Z.; Zheng, M.; Liu, Y. MiR-122 modification enhances the therapeutic efficacy of adipose tissue-derived mesenchymal stem cells against liver fibrosis. *J Cell Mol Med* **2017**, *21*, 2963-2973, doi:10.1111/jcmm.13208.
36. Zhang, B.; Wang, M.; Gong, A.; Zhang, X.; Wu, X.; Zhu, Y.; Shi, H.; Wu, L.; Zhu, W.; Qian, H.; et al. HucMSC-Exosome Mediated-Wnt4 Signaling Is Required for Cutaneous Wound Healing. *Stem Cells* **2015**, *33*, 2158-2168, doi:10.1002/stem.1771.
37. Chew, J.R.J.; Chuah, S.J.; Teo, K.Y.W.; Zhang, S.; Lai, R.C.; Fu, J.H.; Lim, L.P.; Lim, S.K.; Toh, W.S. Mesenchymal stem cell exosomes enhance periodontal ligament cell functions and promote periodontal regeneration. *Acta Biomater* **2019**, *89*, 252-264, doi:10.1016/j.actbio.2019.03.021.
38. Mao, Q.; Nguyen, P.D.; Shanti, R.M.; Shi, S.; Shakoori, P.; Zhang, Q.; Le, A.D. Gingiva-Derived Mesenchymal Stem Cell-Extracellular Vesicles Activate Schwann Cell Repair Phenotype and Promote Nerve Regeneration. *Tissue Eng Part A* **2019**, *25*, 887-900, doi:10.1089/ten.TEA.2018.0176.
39. Tomasoni, S.; Longaretti, L.; Rota, C.; Morigi, M.; Conti, S.; Gotti, E.; Capelli, C.; Introna, M.; Remuzzi, G.; Benigni, A. Transfer of growth factor receptor mRNA via exosomes unravels the regenerative effect of mesenchymal stem cells. *Stem Cells Dev* **2013**, *22*, 772-780, doi:10.1089/scd.2012.0266.
